# Direct Restorative Treatment of Missing Maxillary Laterals with Composite Laminate Veneer: A Case Report

**DOI:** 10.2174/1874210600802010093

**Published:** 2008-06-13

**Authors:** Bora Bagis, Elif Aydoğan, Yildirim H. Bagis

**Affiliations:** 1 Department of Prosthodontics, Faculty of Dentistry, Karadeniz Technical University, Trabzon, Turkey; 2 Department of Restorative Dentistry, Faculty of Dentistry, Ankara University, Ankara, Turkey

## Abstract

This clinical report describes a direct composite laminate veneer restoration of the maxillary anterior teeth in one chair time to produce a better esthetic appearance in a patient with diastemata and missing laterals.

## INTRODUCTION

The maxillary lateral incisor is the second most common congenitally absent tooth [1]. Anomalies in form, color, and position of the anterior teeth cause considerable esthetic problems [2]. Fixed dental prostheses are preferred when treating patients with such anomalies, although the disadvantages of tooth preparation, time of fabrication, and the cost of the restoration sometimes lead the clinician to try another type of restoration. Composite laminate veneers may be an alternative method for these patients. Laminate veneers have become popular as a less invasive and more conservative treatment to provide a more pleasing appearance of the anterior teeth [3]. The long-term clinical success of laminate veneer restorations depends on patient selection, treatment planning, and adhesive bonding techniques [2].

## CLINICAL REPORT

A 29-year-old man with congenitally missing permanent maxillary laterals and diastemata in both the maxilla and mandibula was referred to the Department of Prosthetic Dentistry in Karadeniz Technical University for evaluation and treatment. A detailed dental and medical history was obtained from the patient. Clinical and radiographic examination identified Angle Class 2 Division 1 dental relationship and multiple diastemata. The absence of maxillary laterals was also confirmed by panoramic radiographic examination. The periodontal health status of the patient was within the accepted limitations, and the teeth were without caries. The patient refused to have more chair time treatments or to pay a high cost for the restoration. A treatment plan was developed to improve the patient’s appearance with a direct composite laminate veneer.

The patient refused to use rubber dam during the treatment period to feel relaxed in the dentist chair. First, the maxillary central and canine teeth were dried and prevented from contact with saliva using cotton tampons. The total-etch bonding system was preferred to prepare the surfaces of the teeth because it was deemed necessary to etch the enamel and provide higher bonding strength for the composite resin. Before applying the bonding agent (Single Bond 2, 3M ESPE, St Paul, MN, USA), all of the surfaces to be restored were etched with 35% phosphoric acid gel for 30 seconds. Care was taken to rinse the etchant gel completely for 30 seconds, and the teeth were then air-dried. After this etching procedure, bonding agents were applied to the etched surfaces with brushes, air blow-dried with oil-free air spray, and then cured with an LED curing unit (Elipar FreeLight 2 Led Curing Light, 3M ESPE St Paul, MN, USA) for 20 seconds. The light output of the curing unit was measured with a radiometer (Hilux Ledmax Light Curing Meter, Benlioglu Dental Inc, Ankara, Turkey) as 1100-1200 mW/cm^2^ to confirm that the device was working properly before the curing stage. The buildups were formed with hybrid resin composite (Filtek Z-250, 3M ESPE, St Paul, MN, USA). Composite was placed using an incremental technique, and specific attention was given to the contouring of the marginal finish line of the restorations. The contacts and proximal side of the restorations were formed with celluloid bands. The mandibular central and lateral incisors were restored using the same technique. The maxillary canine teeth were formed in the shape of the lateral teeth, but the distal part of the canine teeth was not restored with composite resin. Gingival finishing lines were examined, and the occlusion was controlled. The final resin composite restorations were polymerized for 40 seconds in the buccal and palatinal or lingual direction.

The restorations were polished with polishing discs (Sof-lex, 3M ESPE St Paul, MN, USA) according to the manufacturer’s instructions. The patient was instructed about how to care for his teeth and protect them from trauma. The patient was examined at three-month intervals for one year. There was no gingival inflammation, erythema, or bleeding, and oral hygiene remained good. No fracture or notable discoloration of the restoration was observed during the follow-up.

## DISCUSSION

Missing maxillary lateral incisors is one of the most important esthetic problems encountered by clinicians asked to improve the smile of their patients. In patients with insufficient space for the prosthetic treatment or malposition of the canines, an interdisciplinary approach is the best choice for treatment [4]. Canines can be moved distally into the correct position for occlusion with orthodontic treatment. The missing lateral incisors can be formed prosthodontically.

Composite laminate, porcelain laminate, metal ceramic crowns, all-ceramic crowns, and fiber-reinforced composite bridges are typical prosthetic treatment alternatives for such patients. The most conservative prosthetic treatment plan would be to place two dental implants for the lateral incisors or to make a fiber-reinforced composite bridge.

In this case, the patient refused orthodontic treatment and dental implants because of the high cost and long duration of treatment. Because of his refusal of orthodontic treatment, there was insufficient space for dental implants. Fiber-reinforced composite bridges might have been an alternative treatment for this patient, but the possible need to prepare the teeth for fiber restoration discouraged the patient and led us to construct the composite laminate veneer restoration. This composite laminate restoration without tooth preparation allowed the patient to see the appearance of his anterior teeth after prosthetic treatment. It was made as a provisional restoration to meet the patient’s esthetic needs without any tooth preparation.

Canines are 1.2 mm wider than the lateral incisors that they are replacing [5,6]. The canine teeth were formed in the lateral teeth shape, but the distal part of the canine teeth was not restored with the composite resin to prevent over shaping of the tooth in the frontal smiling view.

The advantages of the direct laminate technique are its low cost, that the restoration may be evaluated as a reversible treatment procedure, and that the restoration may be repaired intraorally [7]. Although composite resins are esthetic and easy to manipulate, they have some undesired properties such as staining, microleakage, low abrasion resistance, and plaque accumulation, so they are more appropriate to use for anomalies limited with enamel and as provisional restorations [3]. Drinking hot coffee, carbonated beverages, or alcohol may increase discoloration [8]. Increasing the particle size of the resin by decreasing the proportion of organic filler matrix can decrease the change in color [9]. Hybrid composite resins were used in this case because they have good mechanical resistance and can be polished. Light-cured hybrid composite resins are both esthetic and easy to manipulate. [10,11]. In the twelve-month follow-up, there was no remarkable color change, fracture, or damage of the composite restorations. Periodontal tissues remained healthy, and there was no plaque accumulation on the gingival side of the restorations.

An indirect technique allows the clinician to overcome some of the limitations of direct composite resin restorations, such as control of the marginal fit, proximal contacts, anatomic form, color matching, polymerization contraction, and wear resistance [12]. Nevertheless, the indirect technique requires more chair time, and this procedure may lead both the dentist and the patient to choose the direct technique. In addition, an indirect technique may reduce the potential neurotoxic effects of direct composite resin restorations related to incomplete polymerization of a greater than 2 mm incremental addition [13], which we used to avoid incomplete polymerization. Two clinical studies with a follow-up of 10 years or more showed similar rates of failure (16% to 20%) in direct and indirect composite techniques [14,15].

The direct composite laminate technique has become more effective because of improvements in adhesive dentistry. Most manufacturers of dental adhesives offer both a total-etch adhesive and a self-etch adhesive. The highest mean bond strengths to enamel were obtained with total-etch adhesives [16]. In this patient, a total-etch adhesive was used instead of a self-etch adhesive to obtain better bonding to enamel.

Porcelain laminate veneers, metal ceramic crowns, and all-ceramic crowns are expensive and need tooth preparation. These kinds of restorations also take a long time and are irreversible. Based on this knowledge, a direct composite laminate technique may be an important choice for treatment compared with other fixed dental prostheses. Fiber-rein-forced composite bridges can be a good alternative to conventional prosthetic techniques [17].

## SUMMARY

This clinical report describes a direct composite laminate technique for restoring the anterior teeth of a patient with diastemata and missing maxillary laterals. These restorations are conservative and provide a one-chair time esthetic treatment alternative for anterior teeth. Although no remarkable problems were found in the examined follow-up, these kinds of restorations should be evaluated for short-term or provisional restoration.

## Figures and Tables

**Fig. (1):**
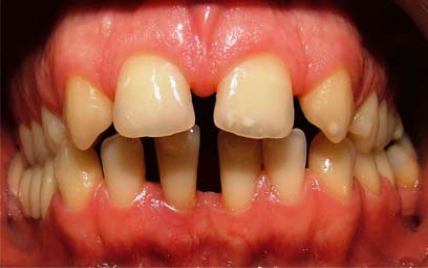
Intraoral view of anterior diastemata before treatment

**Fig. (2):**
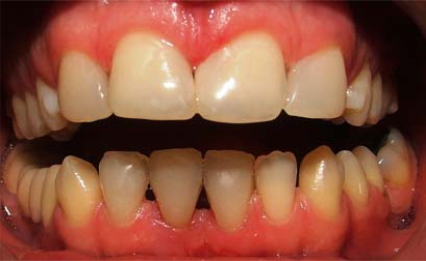
Intraoral view of anterior diastemata after treatment.

**Figs. (3 and 4):**
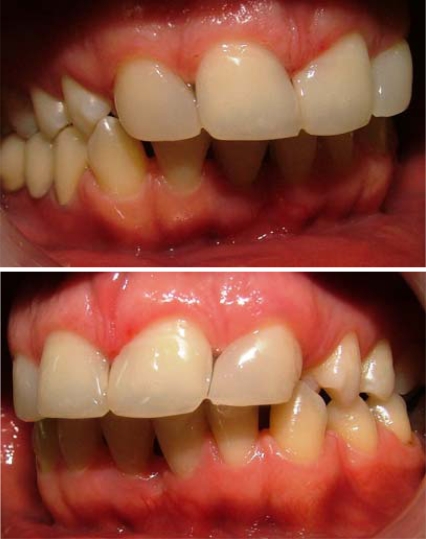
Lateral view after treatment

**Figs. (5 and 6):**
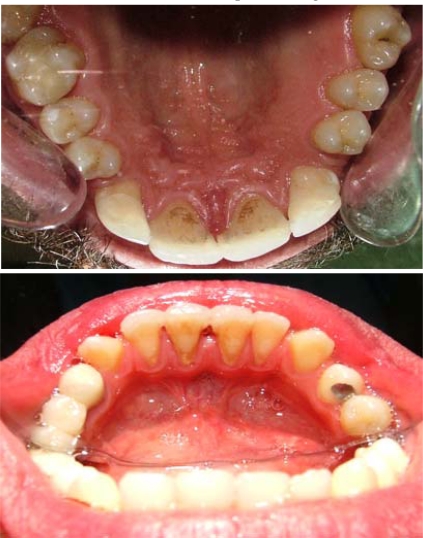
Occlusal view after treatment.
